# Overall survival in patients with lung adenocarcinoma harboring “niche” mutations: an observational study

**DOI:** 10.18632/oncotarget.27472

**Published:** 2020-02-04

**Authors:** Beatrice Aramini, Federico Banchelli, Stefania Bettelli, Samantha Manfredini, Roberto D’Amico, Valentina Masciale, Massimo Pinelli, Margherita Moretti, Alessandro Stefani, Federica Bertolini, Massimo Dominici, Uliano Morandi, Antonino Maiorana

**Affiliations:** ^1^ Division of Thoracic Surgery, Department of Medical and Surgical Sciences, University of Modena and Reggio Emilia, Modena, Italy; ^2^ Division of Oncology, Department of Medical and Surgical Sciences, University of Modena and Reggio Emilia, Modena, Italy; ^3^ Center of Statistics, Department of Medical and Surgical Sciences, University of Modena and Reggio Emilia, Modena, Italy; ^4^ Institute of Pathology, Department of Medical and Surgical Sciences, University of Modena and Reggio Emilia, Modena, Italy; ^5^ Division of Plastic Surgery, Department of Medical and Surgical Sciences, University of Modena and Reggio Emilia, Modena, Italy

**Keywords:** somatic mutations, non-small cell lung cancer (NSCLC), lung cancer treatment, overall survival, target therapy

## Abstract

**Objective:** In addition to the most common somatic lung cancer mutations (i. e., KRAS and EGFR mutations), other genes may harbor mutations that could be relevant for lung cancer. We defined BRAF, c-MET, DDR2, HER2, MAP2K1, NRAS, PIK3CA, and RET mutations as “niche” mutations and analyzed. The aim of this retrospective cohort study was to assess the differences in the overall survival (OS) of patients with lung adenocarcinoma harboring niche somatic mutations.

**Results:** Data were gathered for 252 patients. Mutations were observed in all genes studied, except c-MET, DDR2, MAP2K1, and RET. The multivariable analysis showed that 1) niche mutations had a higher mortality than EGFR mutations (HR = 2.3; 95% CI = 1.2–4.4; *p* = 0.009); 2) KRAS mutations had a higher mortality than EGFR mutations (HR = 2.5; 95% CI = 1.4–4.5; *p* = 0.003); 3) niche mutations presented a similar mortality to KRAS mutations (HR = 0.9; 95% CI = 0.6–1.5; *p* = 0.797).

**Methods:** Three cohorts of mutations were selected from patients with lung adenocarcinoma and their OS was compared. Mutations that were searched for, were 1) BRAF, c-MET, DDR2, HER2, MAP2K1, NRAS, PIK3CA, and RET; 2) K-RAS; and 3) EGFR. Differences in OS between these three cohorts were assessed by means of a multivariable Cox model that adjusted for age, sex, smoking habits, clinical stages, and treatments.

**Conclusions:** Niche mutations exhibited an increased risk of death when compared with EGFR mutations and a similar risk of death when compared with KRAS mutations.

## INTRODUCTION

In the last century, carcinoma of the lung has progressed from an uncommon and obscure disease to the most common cancer in the world, and the most common cause of death from cancer. In 2018, new cases of lung cancer in the US were estimated at 121,680 for men and at 112,350 for women, for a total of 234,030 cases, representing the equivalent of 641 cases of lung cancer diagnosed each day [1, 2]. Many gene mutations have previously been identified in several solid tumors [3, 4]. For lung cancer, in particular, the most common mutations found are in the epidermal growth factor receptor (EGFR) gene and the K-Raf kinase gene family (KRAS) [5]. In addition to these somatic mutations, which are the most frequent, other mutations in several genes have been discovered, including BRAF, c-MET, DDR2, HER2, MAP2K1, NRAS, PIK3CA, and RET mutations [6, 7]. The main feature that characterizes these “niche” mutations is their low prevalence in the population of patients with lung cancer when compared with KRAS and EGFR. In the present study, we identified these “niche mutations” on the basis of a population prevalence that was equal to or less than 10% based on literature data [8–10]. In particular, according to recently published data concerning 14 healthcare institutions in the US, the prevalence for these mutations was as follows: KRAS 25%, EGFR 23%, HER2 2.7%, BRAF 2.6%, PIK3CA 0.8%, NRAS 0.7%, c-MET 0.7%, and MAP2K1 0.3% [8]. Moreover, the prevalence of the RET mutation in adenocarcinoma was estimated to be 1.7% [9], and the prevalence of DDR2 mutation in lung cancer was 2.2% [3, 10]. Overall, the KRAS mutation was considered to be the most lethal mutation, exhibiting the worst prognosis [3–5]. Nevertheless, the potential aggressiveness of niche mutations remains unclear [6, 8].

With regard to treatment, discoveries of gene mutations have allowed the development of targeted therapies, which are considered more effective for survival than chemotherapy in patients with advanced mutated disease [11].

The purpose of this observational retrospective cohort study was to assess differences in overall survival (OS) rates in patients with histologically diagnosed lung adenocarcinoma harboring either the most frequently detected or niche somatic mutations, to highlight new aspects to consider for prognosis and treatment. Considering the potential aggressiveness of niche mutations in this context, the technological advances of next-generation sequencing (NGS), which is currently used in clinical practice, represents a precise approach to identifying a large panel of mutations in oncologic patients [12].

## RESULTS

### Description of patients

Data were gathered from 252 patients. The clinical and demographic characteristics of the studied patients are reported in Table 1. The average age was 67.3 ± 9.9 years, 63.5% of patients were male, and 82.5% of them were smokers. The majority of patients had been diagnosed with stage IV (63.1%) or III (19.0%) cancer according to the TNM (8th Edition). Patients were divided into three groups as follows: 42 (16.7%) patients in the niche mutations group, 155 (61.5%) in the KRAS group, and 55 (21.8%) in the EGFR group. We found differences in distributions of gender and smoking habits among the three cohorts. Male patients and smokers were more common in the KRAS group (74.8% males and 90.3% smokers) than in the niche mutations group (61.9% and 81.0%) or the EGFR mutation group (32.7% and 61.8%) (*p* = 0.000 and *p* = 0.000). Moreover, the cohorts received different treatments because the frequency of chemotherapy and target therapy was not similar between the groups. In the KRAS groups, chemotherapy was performed on 79.4% of the patients, whereas chemotherapy was performed on 69.0% and 38.2% of patients in the niche mutations and EGFR mutations groups, respectively (p = 0.000). However, target therapy was more common in the EGFR mutation group (52.7%) than in the KRAS mutation (0.0%) and niche mutations (7.1%) groups (*p* = 0.000). The three cohorts were similar in terms of the remaining characteristics (i. e., age (*p* = 0.376), TNM stage (*p* = 0.078), surgery (*p* = 0.940), radiotherapy (p = 0.462), and immunotherapy (*p* = 0.409). The most common first-line treatment was chemotherapy for the KRAS and niche mutations cohorts (53.5% and 42.9% of patients, respectively), whereas in the EGFR cohort, target therapy was more common (29.1% of patients). Eight (3.2%) patients did not undergo any treatment after diagnosis. The characteristics of patients harboring every single-niche mutation are reported in Supplementary Table 1.

**Table 1:** Patients characteristics at primary diagnosis and treatments after diagnosis

		KRAS (*n* = 155)	EGFR (*n* = 55)	Niche mutations (*n* = 42)	All patients (*n* = 252)	*p*-value
**Patients characteristics at primary diagnosis**						
Age – Years	mean ± SD	67.4 ± 9.4	65.9 ± 12.1	68.8 ± 8.4	67.3 ± 9.9	0.376
Gender – M	*n* (%)	116 (74.8%)	18 (32.7%)	26 (61.9%)	160 (63.5%)	0.000
Smoker – Yes	*n* (%)	140 (90.3%)	34 (61.8%)	34 (81.0%)	208 (82.5%)	0.000
Stage - I	*n* (%)	9 (5.8%)	8 (14.5%)	1 (2.4%)	18 (7.1%)	0.078
Stage – II	*n* (%)	18 (11.6%)	5 (9.1%)	4 (9.5%)	27 (10.7%)	
Stage – III	*n* (%)	27 (17.4%)	7 (12.7%)	14 (33.3%)	48 (19.0%)	
Stage - IV	*n* (%)	101 (65.2%)	35 (63.6%)	23 (54.8%)	159 (63.1%)	
**Treatments after diagnosis**						
Surgery	*n* (%)	42 (27.1%)	16 (29.1%)	12 (28.6%)	70 (27.8%)	0.940
RT	*n* (%)	75 (48.4%)	27 (49.1%)	25 (59.5%)	127 (50.4%)	0.462
CT	*n* (%)	123 (79.4%)	21 (38.2%)	29 (69.0%)	173 (68.7%)	0.000
IMT	*n* (%)	36 (23.2%)	8 (14.5%)	8 (19.0%)	52 (20.6%)	0.409
TT	*n* (%)	0 (0.0%)	29 (52.7%)	3 (7.1%)	32 (12.7%)	0.000
**First line treatment**						
Surgery	*n* (%)	35 (22.6%)	12 (21.8%)	9 (21.4%)	56 (22.2%)	0.000
RT	*n* (%)	27 (17.4%)	8 (14.5%)	7 (16.7%)	42 (16.7%)	
CT	*n* (%)	83 (53.5%)	12 (21.8%)	18 (42.9%)	113 (44.8%)	
IMT	*n* (%)	6 (3.9%)	6 (10.9%)	3 (7.1%)	15 (6.0%)	
TT	*n* (%)	0 (0.0%)	16 (29.1%)	2 (4.8%)	18 (7.1%)	
No treatment	*n* (%)	4 (2.6%)	1 (1.8%)	3 (7.1%)	8 (3.2%)	

RT: Radiotherapy; CT: Chemotherapy; IMT: Immunotherapy; TT: Target Therapy; SD: standard deviation.

### Description of mutations

The observed distribution of single mutations is reported in Table 2. The most frequent mutation was found in the KRAS gene (found in 63.5% of the patients), whereas EGFR gene mutations were observed in 22.6% of the patients. In the niche mutations group, BRAF gene mutations were observed in 6.7% of the patients, whereas HER2 mutations were observed in 4.4% of the patients, PIK3CA gene mutations were detected in 4.0% of the patients and NRAS in 1.6%. No c-MET, DDR2, MAP2K1, or RET mutations were identified in any patient included in the study. Seven patients (2.8%) showed concomitant mutations in two genes and were all classified in the niche mutations cohort. The specific codons, exons, and amino acid alterations identified are reported in detail in Table 2.

**Table 2:** Mutations, codons, exons and amino acid alterations identified in the study population

Frequent mutations			Niche mutations			Overall		
	*n*	%		*n*	%		*n*	%
KRAS single mutation	155	61.5%	BRAF single mutation	16	6.3%	KRAS	160	63.5%
Codon 12	136	54.0%	Codon 469	4	1.6%	EGFR	57	22.6%
G12C	70	27.8%	Codon 600	6	2.4%	BRAF	17	6.7%
G12V	27	10.7%	Codon 466	3	1.2%	HER2	11	4.4%
G12D	22	8.7%	HER2 single mutation	10	4.0%	PIK3CA	10	4.0%
G12A	15	6.0%	Codon 775 & 776 ins YVMA	7	2.8%	NRAS	4	1.6%
Codon 13	13	5.2%	PIK3CA single mutation	6	2.4%	Concomitant mutations	7	2.8%
G13C	8	3.2%	Codon 542	1	0.4%			
Codon 61	6	2.4%	Codon 545	1	0.4%			
EGFR single mutation	55	21.8%	NRAS single mutation	3	1.2%			
Exon 19	16	6.3%	Codon 61	1	0.4%			
Codon del 746 & 750	14	5.6%	Concomitant mutations	7	2.8%			
Exon 21	17	6.7%	KRAS & PIK3CA	2	0.8%			
Codon 858	13	5.2%	EGFR & PIK3CA	2	0.8%			
Exon 18	4	1.6%	KRAS & BRAF	1	0.4%			
Exon 20	3	1.2%	KRAS & NRAS	1	0.4%			
Exons 20 & 21	5	2.0%	KRAS & HER2	1	0.4%			
Exons 19 & 20	4	1.6%						
Exons 18 & 20	2	0.8%						
Exons 19 & 21	1	0.4%						

Notes: Counts at different levels of details may differ. All identified exons and codons were reported and only amino acids alterations with absolute frequency ≥ 5 are shown. Mutations for which the codon was not identified are not considered in the table.

### Mortality rates

Over an average follow-up period of 1.3 (range 0.1 to 4.8) years, there were 168 (66.7%) deaths, 167 of which were cancer-related. In the niche mutations cohort, 29 (69.0%) patients died, in the KRAS gene cohorts 109 (70.3%) died, and in the EGFR gene cohort 30 (54.5%) died.

**Figure 1:**
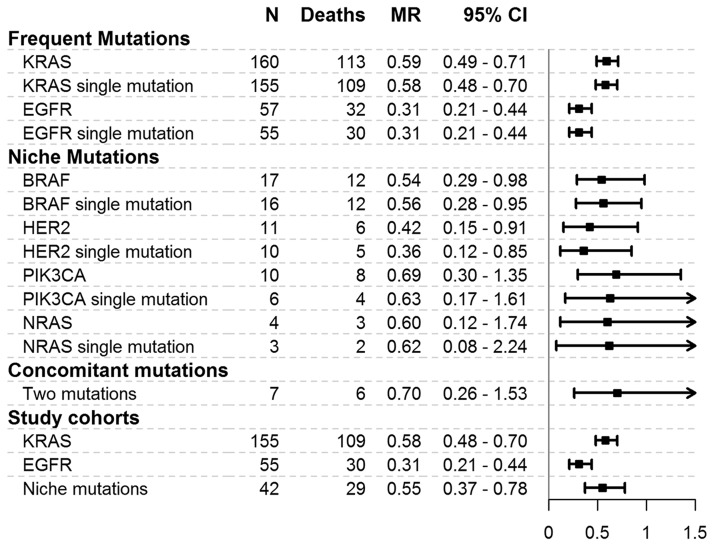
Mortality rates for mutations and cohorts considered in the study. MR = mortality rate, expressed as the number of deaths per person-year. 95% CI = 95% confidence interval. N = number of patients. The plot reports mortality rates and 95% confidence intervals.

MRs for each gene mutation and cohort considered are reported in Figure 1. Patients with KRAS mutations had an MR of 0.59 (95% CI = 0.49–0.71) deaths per year, whereas the MRs were 0.31 (0.21–0.44) for EGFR gene patients, 0.54 (0.29–0.98) for BRAF gene patients, 0.42 (0.15–0.91) for HER2 gene patients, 0.69 (0.30–1.35) for PIK3CA gene patients, and 0.60 (0.12–0.74) for NRAS gene patients. The subjects with two mutations presented an MR of 0.70 (0.26–1.53). The mortality was higher in the KRAS gene cohort (MR = 0.58, 95% CI = 0.48–0.70) and in the niche mutations cohort (MR = 0.55, 95% CI = 0.37–0.78) than in the EGFR gene cohort (MR = 0.31, 95% CI = 0.21–0.44).

### Differences in overall survival

**Figure 2:**
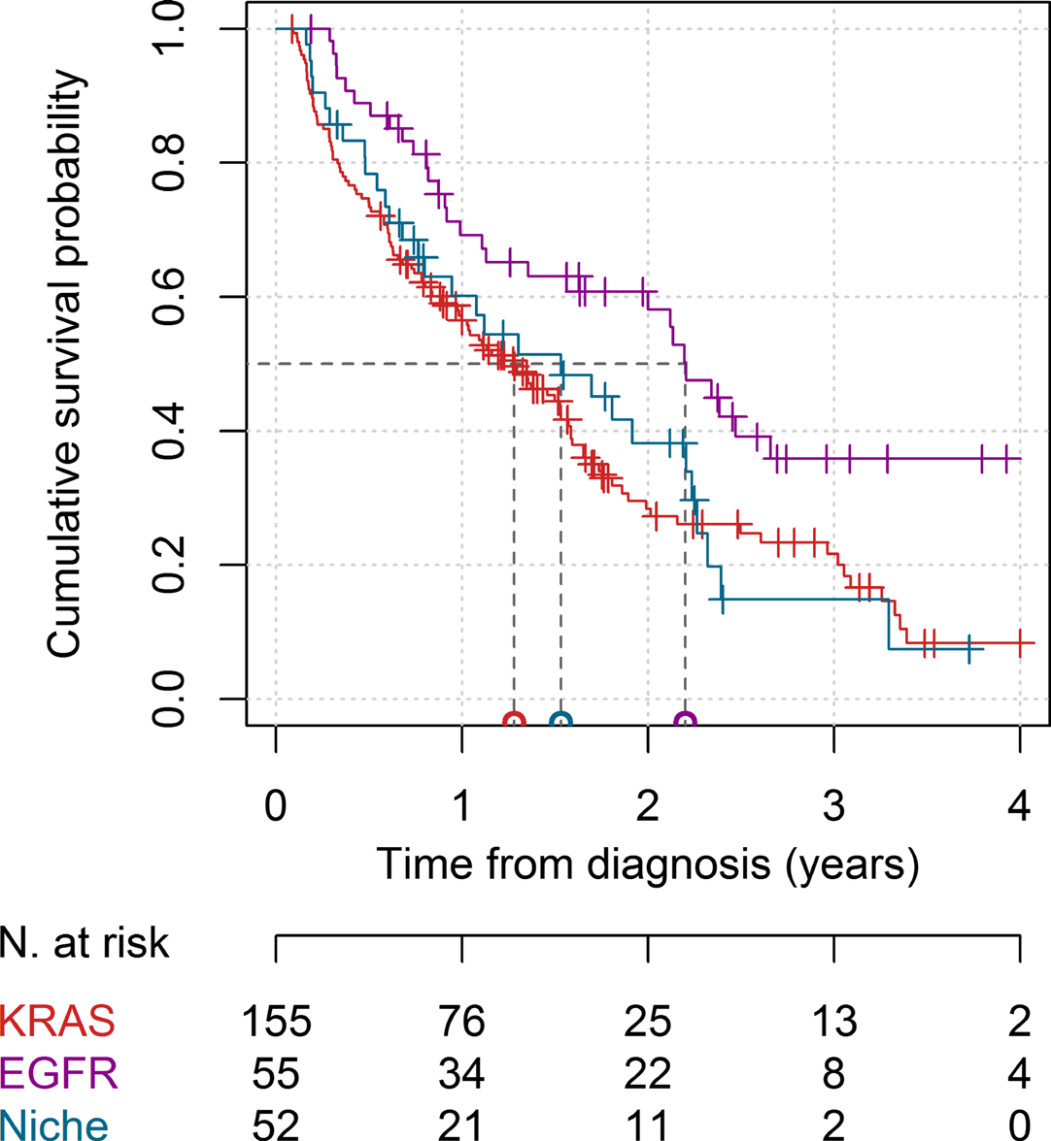
Survival curves in the KRAS, EGFR, and niche mutations cohorts. The red line represents the KRAS cohort, the purple line represents the EGFR cohort and the blue line represents the niche mutations cohort. The median survival times for the three cohorts are reported on the horizontal axis. The number of patients at risk at 0, 1, 2, 3, and 4 years from baseline is reported. Thick marks represent subjects lost to follow-up.

The survival curves for the three cohorts are shown in Figure 2. The median OS was measured as 1.5 years (95% CI = 1.2–1.8) for the whole population, 1.5 years (95% CI = 0.8–2.3) for the niche mutations cohort, 1.3 (95% CI = 1.0–1.6) years for the KRAS gene cohort, and 2.2 years (95% CI not estimable) for the EGFR gene cohort. The cumulative survival probability at 1 year from diagnosis was measured as 56.5% (95% CI = 49.1–65.0%) for the KRAS gene mutation cohort, as 60.1% (46.6–77.6%) for the niche mutations cohort, and as 69.2% (57.7–83.1%) for the EGFR gene mutations cohort, whereas at 2 years these values were measured as 28.4% (21.2–38.0%), 38.2% (24.9–58.6%), and 58.2% (45.8–73.8%), respectively.

**Figure 3:**
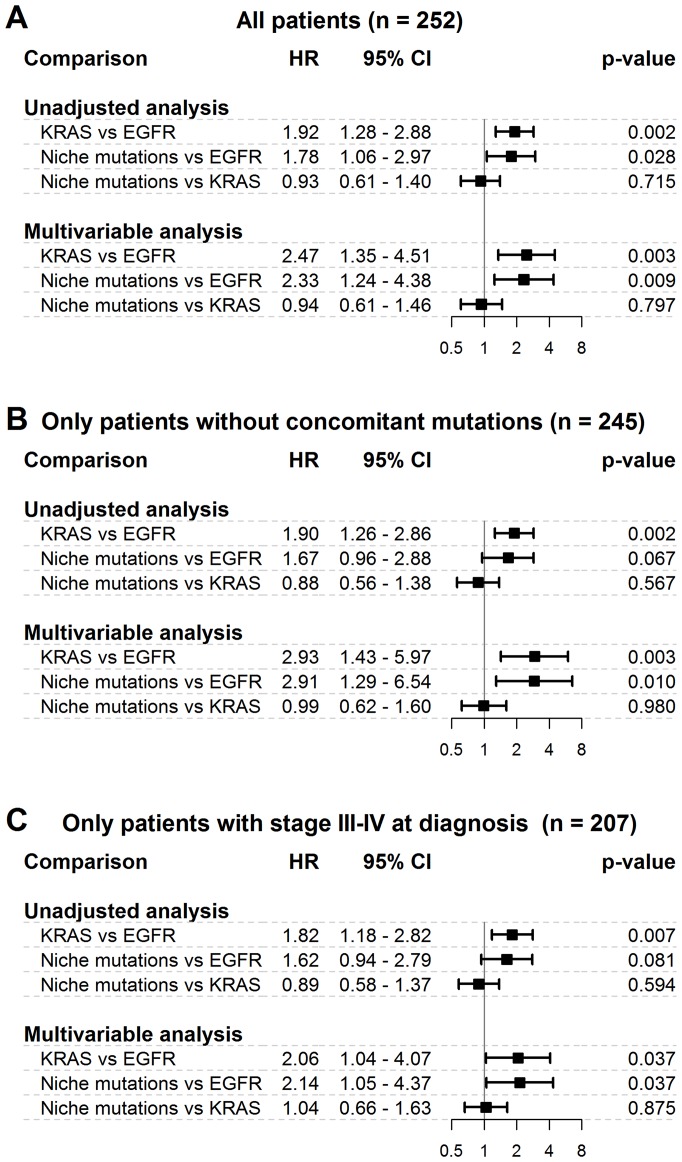
Differences in overall survival among niche mutations cohort and frequent mutations cohorts. HR = hazard ratio; 95% CI = 95% confidence interval. (**A**) = main analysis - all patients. (**B**) = secondary analysis – only patients without concomitant mutations. (**C**) = secondary analysis – only patients with stage III–IV at diagnosis. Results from both unadjusted and multivariable Cox regression analysis are reported. The multivariable model was adjusted for age, sex, smoking habits, stage and treatments. The plots report hazard ratios and 95% confidence intervals.

The differences in OS observed between the three cohorts of interest are reported in Figure 3 from the unadjusted and multivariable regression analyses. According to the multivariable analysis that adjusted for age, sex, smoking habits, stage, and treatments, we observed that 1) patients with niche mutations had a higher risk of death than patients with EGFR gene mutations (HR = 2.3; 95% CI = 1.2–4.4; *p* = 0.009); 2) patients with KRAS gene mutations had a higher risk of death than patients with EGFR gene mutations (HR = 2.5; 95% CI = 1.4–4.5; *p* = 0.003); 3) patients with niche mutations presented a risk of death similar to patients with KRAS gene mutations (HR = 0.9; 95% CI = 0.6–1.5; *p* = 0.797). These results did not change when patients with concomitant mutations were excluded or when patients with stage I or II diagnoses were excluded (Figure 3). The associations between all the recorded independent variables and the risk of death, as assessed by the Cox multivariable model, are reported in Supplementary Table 2. The proportional hazards assumption was verified for all HRs of interest (data not shown).

## DISCUSSION

Our study reports a retrospective analysis on OS rates observed in patients with lung adenocarcinoma harboring somatic mutations either in EGFR and KRAS genes, or in less frequently studied genes, such as BRAF, PIK3CA, NRAS, and HER2 (niche mutations). In our work, we analyzed and described the clinical characteristics of the three cohorts of patients. From our data, male subjects and smokers were more common in the KRAS group than in the niche mutations and EGFR groups. Furthermore, target therapies were more common in the EGFR group, whereas chemotherapy was more common in the KRAS and niche mutations cohorts. This is justified by the fact that no specific target treatment has yet been developed for KRAS and niche mutations.

Regarding treatments given after diagnosis, we observed that our cohorts of patients were treated as previously described for general mutations [15, 16]. This is likely because niche mutations are poorly understood, especially in terms of biological characteristics and interactions. However, we believe that the percentage of patients harboring niche mutations suggests further study, in particular with respect to treatment strategies.

In our study, we underlined the importance of niche mutations in terms of OS relative to the most frequent general somatic mutations and for KRAS in particular, which is considered to be the most frequent and aggressive mutation in lung adenocarcinoma. EGFR and KRAS gene mutations are already recorded in the molecular report that an oncologist needs to provide “personalized” therapy [17–20]. In addition to the mutations for which target therapies have been generally successful, for example, for the EGFR gene, there are still mutations without effective therapies, as for the KRAS and niche mutations. The scientific community has agreed on the fact that KRAS is one of the most aggressive mutations in terms of prognosis and survival because it exhibits a high mortality rate and there is no existing targeted treatment available [17, 18]. Our data described a similar OS for KRAS and niche mutations. This aspect is very interesting and needs to be further considered, although little has been published on niche mutations in lung cancer, probably because they are only observed in a small proportion of the population.

We first described a large cohort study analyzing niche mutations in adenocarcinoma of the lung and their prognostic role as compared with the more frequently diagnosed mutations. In the current scientific literature, many studies have described the occurrence of general somatic mutations in NSCLC, although some niche mutations, such as c-MET and RET in adenocarcinoma of the lung have been described in a few short reports [19–22], or in a larger population focusing on a single-niche mutation of PIK3CA [23–25]. We took into consideration different rare mutations observed in BRAF, PIK3CA, HER2, and NRAS detected with a total frequency equal to 16.7% of patients with lung adenocarcinoma harboring somatic mutations, which is not a negligible prevalence if transposed to the entire population. Moreover, niche mutations presented a high risk of death, accounting for 55% of deaths in one person-year.

The most interesting and novel results that came out of this research were that 1) niche mutations showed very similar rates of survival as those of patients with KRAS gene mutation; 2) patients harboring niche or KRAS gene mutations showed a higher risk of mortality than those with EGFR gene mutations.

We know that mutations in the KRAS gene are the most aggressive, and for this reason, we highlighted the importance of evaluating these niche mutations through our results. The life expectancy of these patients was low and seemed to be very similar to that of patients with well-established aggressive general mutations. These patients may benefit from the use of specific inhibitors such as dabrafenib/trametinib, which are used for BRAF v600E mutations [26].

### Limitations

Finally, the present study has some limitations that should be discussed. First, the observational and retrospective nature of this study presents a risk of bias; however, adjustments made to the multivariable analysis protected against confounding bias due to the observed patient characteristics. Nevertheless, other unobserved factors may have biased our results. Second, even if we observed statistically significant differences in the OS between niche mutations and general mutations in a large set of consecutive patients enrolled over 5 years, another limitation relates to the low absolute number of patients with niche mutations that were present. Because of this, it was not possible properly to assess differences in OS between the single-niche mutations. Moreover, because c-MET, DDR2, MAP2K1, and RET gene mutations were not observed in this study, we cannot draw conclusions about the OS of patients harboring these niche mutations.

Taken together, these limitations highlight the need for an extended study to confirm our findings and to reduce further the uncertainty regarding the prognostic role of niche mutations.

## MATERIALS AND METHODS

This retrospective cohort study was carried out in accordance with the STrengthening the Reporting of OBservational studies in Epidemiology (STROBE) statement [13].

### Study population

All patients with lung adenocarcinoma identified at the Modena University Hospital between January 1, 2013 and December 31, 2017, and for whom a molecular analysis of gene mutations was available were eligible for inclusion. Inclusion criteria were histologically diagnosed primary or metastatic adenocarcinoma of the lung, age at primary diagnosis between 18 and 90 years, presence of at least one of BRAF, c-MET, DDR2, EGFR, HER2, KRAS, MAP2K1, NRAS, PIK3CA, and RET gene mutations. Exclusion criteria were patients with wild-type genes, patients with an unknown tumor, squamous cell carcinoma of the lung, large-cell carcinoma of the lung, small-cell lung cancer, and neuroendocrine tumors of the lung.

All patients received standard treatments according to the current guidelines based on clinical stage and other relevant clinical factors.

### Data collection

Data were retrospectively collected based on electronic hospital patient records. The following information was gathered: date of primary diagnosis; age at diagnosis (years); sex (M, F); clinical stage at diagnosis (TNM 8th Edition) [14]; smoking status at diagnosis (yes, no); treatments performed after diagnosis (surgery, chemotherapy, radiotherapy, immunotherapy, target therapy); first-line treatment; presence of BRAF, c-MET, DDR2, EGFR, HER2, KRAS, MAP2K1, NRAS, PIK3CA, or RET gene mutations and related codons; date and cause of death; date of last follow-up.

The somatic mutations that were considered were those detectable by our genotyping platform.

### Molecular analysis

Molecular analysis of gene mutations was performed for patients with advanced lung cancer or for early-stage surgical patients after recurrence.

DNA was extracted from 10 μm thick representative sections cut from formalin-fixed and paraffin-embedded blocks of each tumor sample containing at least 50% tumor cells. Extraction was performed with the QIAamp DNA Mini Kit (Qiagen, Hilden, Germany), and the DNA was quantified with Xpose NGS (Trinean NV, Gentbrugge, Belgium). Mutations were detected using the high-throughput genotyping platform Sequenom MassARRAY^®^ System (Sequenom, San Diego, CA, USA) and the Myriapod^®^ Lung Status kit (Diatech Pharmacogenetics, Italy) following the manufacturers’ protocols. The molecular array allowed for the identification of the mutations of the BRAF, c-MET, DDR2, EGFR, HER2, KRAS, MAP2K1, NRAS, PIK3CA, and RET genes. In brief, 25 ng DNA of each specific primitive tumor was amplified through a multiplex polymerase chain reaction and then incorporated nucleotides were inactivated using shrimp alkaline phosphatase. A single-base extension reaction was performed using extension primers that hybridized immediately adjacent to the mutations and a custom mixture of nucleotides. Salts were removed using a cation exchange resin. Multiplexed reactions were spotted in SpectroCHIP II arrays, and DNA fragments were resolved with matrix-assisted laser desorption/ionization–time-of-flight (MALDI–TOF) using a compact mass spectrometer (Sequenom, San Diego, CA, USA) with a detection limit of 5%. The data were evaluated using MassARRAY Typer analyzer software 4.0, which allowed the identification of mutated alleles by comparing the ratio of the wild-type peak of all suspected mutants.

### Statistical analysis

Descriptive characteristics of the patients at the time of diagnosis were recorded. Continuous variables were recorded as mean ± standard deviation (SD) values, and categorical variables were recorded as absolute and percentage frequencies. Comparisons of group characteristics were drawn using analysis of variance and chi-square tests. OS was defined as the time span from diagnosis to death (all-cause mortality was considered). The OS of patients who were still alive at the end of the follow-up period were treated as censored survival times. The main aim of the analysis was to compare the OS of three cohorts of patients harboring: 1) niche mutations (BRAF, c-MET, DDR2, HER2, MAP2K1, NRAS, PIK3CA, or RET); 2) KRAS mutations; 3) EGFR mutations. The mortality rates (MRs) were calculated as the number of deaths per person-year. The OS curves were calculated using the Kaplan–Meier method. The pairwise differences in OS between the three cohorts of interest were assessed by means of a Cox proportional hazards regression model. The Cox model results were reported as hazard ratios (HRs) with 95% confidence interval (95% CI) from unadjusted analysis (i. e., with the cohort as the only independent variable) and from a multivariable analysis that adjusted for the confounding effects attributable to age (years); sex (M vs F); smoking habits (yes vs no); clinical stage (III, IV vs I–II) at diagnosis; and treatments after diagnosis (surgery, chemotherapy, radiotherapy, immunotherapy, and target therapy – all yes vs no). The proportional hazards assumption was validated by a graphical assessment of scaled Schoenfeld residuals. All statistical analyses were performed with R 3.4.3 statistical software (The R Foundation for Statistical Computing, Wien) at the *p* < 0.05 significance level.

## CONCLUSIONS

We found evidence of a higher risk of death for patients harboring niche mutations relative to patients with EGFR gene mutations, whereas the risk for patients with niche mutations was found to be comparable to that of patients harboring KRAS gene mutations. We may summarize this message in two points: 1. As soon as the molecular biologist is able to identify a niche mutation, it is necessary to highlight this in the patient report to set a targeted treatment that offers a better solution than chemotherapy for the patient; 2. The NGS technique, which is able to select a myriad of genes, is more useful for the oncologist who is conscious of the importance of considering niche mutations.

The correct selection of mutations will be helpful in terms of the greater efficacy of treatment in association with better prognosis and a higher quality of life for oncologic patients.

## SUPPLEMENTARY MATERIALS



## Abbreviations

NSCLC: non-small cell lung cancer
EGFR: epidermal growth factor receptor
BRAF: B-Raf kinase family
KRAS: K-Raf kinase family
c-MET: tyrosine-protein kinase Met
PIK3CA: phosphatidylinositol-4,5-bisphosphate 3-kinase catalytic subunit alpha
NRAS: neuroblastoma RAS viral oncogene homolog
MAP2K1: Mitogen-Activated Protein Kinase Kinase
HER2: human epidermal growth factor receptor 2
DDR2: discoidin domain receptor tyrosine kinase 2
RET: REarranged during Transfection
OS: overall survival
MRs: Mortality rates
NGS: the next-generation sequencing
STROBE: STrenghening the Reporting of Observational studies in Epidemiology
