# Supplementary material for: Overall survival in patients with lung adenocarcinoma harboring “niche” mutations: an observational study
Source: Oncotarget. 2020 Feb 4;11(5):550–9. doi: 10.18632/oncotarget.27472 (PMC7007296; doi:10.18632/oncotarget.27472)
Supplement: Supplementary file 1 [file oncotarget-11-550-s001.pdf]

Overall survival in patients with lung adenocarcinoma harboring "niche" mutations: an observational study

SUPPLEMENTARY MATERIALS

Supplementary Table 1: Patients characteristics at primary diagnosis and treatments after diagnosis for every niche mutations subgroup

		BRAF (*n* = 17)	HER2 (*n* = 11)	PIK3CA (*n* = 10)	NRAS (*n* = 4)
**Patients characteristics at primary diagnosis**					
Age – Years	mean ± SD	68.6 ± 8.9	69.7 ± 11.2	67.7 ± 6.0	69.2 ± 1.7
Gender – M	*n* (%)	12 (70.6%)	5 (45.5%)	8 (80.0%)	1 (25.0%)
Smoker – Yes	*n* (%)	15 (88.2%)	7 (63.6%)	9 (90.0%)	3 (75.0%)
Stage - I	*n* (%)	0 (0.0%)	0 (0.0%)	1 (10.0%)	0 (0.0%)
Stage – II	*n* (%)	1 (5.9%)	1 (9.1%)	2 (20.0%)	0 (0.0%)
Stage – III	*n* (%)	10 (58.8%)	4 (36.4%)	0 (0.0%)	0 (0.0%)
Stage - IV	*n* (%)	6 (35.3%)	6 (54.5%)	7 (70.0%)	4 (100.0%)
**Treatments after diagnosis**					
Surgery	*n* (%)	9 (52.9%)	1 (9.1%)	2 (20.0%)	0 (0.0%)
RT	*n* (%)	8 (47.1%)	6 (54.5%)	8 (80.0%)	3 (75.0%)
CT	*n* (%)	12 (70.6%)	7 (63.6%)	7 (70.0%)	3 (75.0%)
IMT	*n* (%)	4 (23.5%)	2 (18.2%)	1 (10.0%)	1 (25.0%)
TT	*n* (%)	0 (0.0%)	1 (9.1%)	2 (20.0%)	0 (0.0%)
**First line treatment**					
Surgery	*n* (%)	6 (35.3%)	1 (9.1%)	2 (20.0%)	0 (0.0%)
RT	*n* (%)	2 (11.8%)	2 (18.2%)	3 (30.0%)	0 (0.0%)
CT	*n* (%)	7 (41.2%)	5 (45.5%)	3 (30.0%)	3 (75.0%)
IMT	*n* (%)	0 (0.0%)	1 (9.1%)	1 (10.0%)	1 (25.0%)
TT	*n* (%)	0 (0.0%)	1 (9.1%)	1 (10.0%)	0 (0.0%)
No treatment	*n* (%)	2 (11.8%)	1 (9.1%)	0 (0.0%)	0 (0.0%)

RT: Radiotherapy; CT: Chemotherapy; IMT: Immunotherapy; TT: Target Therapy; SD: standard deviation.

Supplementary Table 2: Results of multivariable regression analysis

	Multivariable analysis		
	**HR**	**95% CI**	***p*-value**
KRAS vs. EGFR	2.47	1.35–4.51	0.003
Niche mutations vs. EGFR	2.33	1.24–4.38	0.009
Sex – M vs. F	1.17	0.80–1.71	0.408
Age – +1 year	1.01	0.99–1.02	0.430
Smoker – Yes vs. No	1.45	0.91–2.31	0.118
Stage – III vs. I-II	2.42	1.28–4.57	0.006
Stage – IV vs. I-II	3.40	1.53–7.56	0.003
Surgery – Yes vs. No	0.60	0.31–1.19	0.147
RT – Yes vs. No	1.08	0.78–1.51	0.629
CT – Yes vs. No	0.55	0.37–0.82	0.003
IMT – Yes vs. No	0.59	0.38–0.92	0.019
TT – Yes vs. No	1.00	0.53–1.92	0.993

Notes: HR: hazard ratio; 95% CI: 95% confidence interval; RT: Radiotherapy; CT: Chemotherapy; IMT: Immunotherapy; TT: Target Therapy.
